# miss-SNF: a multimodal patient similarity network integration approach to handle completely missing data sources

**DOI:** 10.1093/bioinformatics/btaf150

**Published:** 2025-04-04

**Authors:** Jessica Gliozzo, Mauricio A Soto Gomez, Arturo Bonometti, Alex Patak, Elena Casiraghi, Giorgio Valentini

**Affiliations:** AnacletoLab, Dipartimento di Informatica “Giovanni Degli Antoni”, Università degli Studi di Milano, Via Giovanni Celoria 18, Milan, 20133, Italy; European Commission, Joint Research Centre (JRC), Ispra, 21027, Italy; AnacletoLab, Dipartimento di Informatica “Giovanni Degli Antoni”, Università degli Studi di Milano, Via Giovanni Celoria 18, Milan, 20133, Italy; Department of Biomedical Sciences, Humanitas University, Via Rita Levi Montalcini 4, Pieve Emanuele (MI), 20072, Italy; Department of Pathology, IRCCS Humanitas Clinical and Research Hospital, Via Alessandro Manzoni 56, Rozzano (MI), 20089, Italy; European Commission, Joint Research Centre (JRC), Ispra, 21027, Italy; AnacletoLab, Dipartimento di Informatica “Giovanni Degli Antoni”, Università degli Studi di Milano, Via Giovanni Celoria 18, Milan, 20133, Italy; Environmental Genomics and Systems Biology Division, Lawrence Berkeley National Laboratory, Berkeley, CA, 94720, United States; Milan Unit, ELLIS—European Laboratory for Learning and Intelligent Systems, Italy; AnacletoLab, Dipartimento di Informatica “Giovanni Degli Antoni”, Università degli Studi di Milano, Via Giovanni Celoria 18, Milan, 20133, Italy; Milan Unit, ELLIS—European Laboratory for Learning and Intelligent Systems, Italy

## Abstract

**Motivation:**

Precision medicine leverages patient-specific multimodal data to improve prevention, diagnosis, prognosis, and treatment of diseases. Advancing precision medicine requires the non-trivial integration of complex, heterogeneous, and potentially high-dimensional data sources, such as multi-omics and clinical data. In the literature, several approaches have been proposed to manage missing data, but are usually limited to the recovery of subsets of features for a subset of patients. A largely overlooked problem is the integration of multiple sources of data when one or more of them are completely missing for a subset of patients, a relatively common condition in clinical practice.

**Results:**

We propose miss-Similarity Network Fusion (miss-SNF), a novel general-purpose data integration approach designed to manage completely missing data in the context of patient similarity networks. miss-SNF integrates incomplete unimodal patient similarity networks by leveraging a non-linear message-passing strategy borrowed from the SNF algorithm. miss-SNF is able to recover missing patient similarities and is “task agnostic”, in the sense that can integrate partial data for both unsupervised and supervised prediction tasks. Experimental analyses on nine cancer datasets from The Cancer Genome Atlas (TCGA) demonstrate that miss-SNF achieves state-of-the-art results in recovering similarities and in identifying patients subgroups enriched in clinically relevant variables and having differential survival. Moreover, amputation experiments show that miss-SNF supervised prediction of cancer clinical outcomes and Alzheimer’s disease diagnosis with completely missing data achieves results comparable to those obtained when all the data are available.

**Availability and implementation:**

miss-SNF code, implemented in R, is available at https://github.com/AnacletoLAB/missSNF.

## 1 Introduction

Precision medicine (PM) aims to improve disease prevention, diagnosis, prognosis, and management by enabling more effective stratification of patient sub-populations and the development of targeted therapies (Johansson *et al.* 2023). These advancements are fueled by the increasing availability of large-scale, multimodal patient data (e.g. multi-omics, clinical, demographic, and wearable device data), which collectively provide a holistic view of disease states (Athieniti and Spyrou 2023). In particular, integrating multi-omics data enables the comprehensive biomolecular characterization of individuals, which is crucial to study complex diseases like cancer, cardiovascular disorders, and others (Karczewski and Snyder 2018, Olivier *et al.* 2019).

Multi-view data integration has been applied especially in the context of multi-view clustering (Yang and Wang 2018), for instance to integrate different views of images (Zeng *et al.* 2024), also considering missing data (Gong *et al.* 2022, Yang *et al.* 2023), or by jointly learning data restoration, data denoising, and clustering (Wang *et al.* 2024).

The integration of multi-omics data raises specific challenges, addressed in the literature through four main strategies (Gliozzo *et al.* 2022): (1) Input data-fusion methods apply factorization techniques to embed patients into an integrated patient space for clustering or classification. A notable example is MOFA+, which achieves impressive results using an efficient variational inference approach (Argelaguet *et al.* 2020). (2) Output-fusion approaches independently process each omics view before combining results across views. (3) Task-specific integrated patient embeddings, such as graph neural networks (GNNs), showed promising results (Ryan *et al.* 2024), but they often lack generalization across diverse tasks, require large datasets—rare in the medical domain—and are affected by class imbalances inherent in medical data. (4) Patient similarity network (PSN)-fusion techniques construct unimodal PSNs for each omics view, where nodes represent patients and edges encode similarities between omics profiles. These unimodal PSNs are then fused into an integrated PSN space, supporting clustering and classification tasks. PSN-based methods, like NEMO (Rappoport and Shamir 2019) and the widely used Similarity Network Fusion (SNF) (Wang *et al.* 2014), have demonstrated robust performance across multi-omics tasks, with additional benefits such as interpretability and privacy preservation (Pai and Bader 2018, Dai *et al.* 2020, Gliozzo *et al.* 2020).

In this work, we focus on the multi-omics integration of PSNs with completely missing data sources. In other words, we address the multi-view integration of graphs where nodes represent patients and edges represent biomolecular or clinical similarities, and one or more data sources (e.g. transcriptomics or methylomics data) are entirely missing for some patients.

Several methods have been proposed for managing multi-omics missing data (Liu *et al.* 2023, Ren *et al.* 2024), i.e. to recover subsets of features not available for subsets of patients, but in clinical practice it is quite common that some data sources are completely missing for subsets of patients, and this represents an open problem in the context of multi-omics and clinical data integration (Gliozzo *et al.* 2022). Indeed, although state-of-the-art fusion methods are effective for disease subtype identification, biomarker discovery, and prognosis prediction, most cannot handle incomplete, or “partial”, datasets (Gliozzo *et al.* 2022, Flores *et al.* 2023) where one or more data sources are entirely missing for some patients (Supplementary Fig. S7). Recently, a deep generative model for multi-omics integration with completely missing data was proposed (Ashuach *et al.* 2023). However, it is specifically designed for identifying cell sub-populations by fusing scRNA-seq and scATAC-seq data in a single-cell context, making its application to a PSN scenario less feasible (Ashuach *et al.* 2023).

While some methods, such as MOFA+ and NEMO, can process partial datasets, their applications are often limited to clustering or classification tasks. Furthermore, MOFA+ relies on a stochastic variational inference framework optimized via gradient descent, which achieves optimal results and stable convergence only when the number of available samples is significantly larger than the number of features. This is further supported by results reported in Gliozzo *et al.* (2025), which indicate that when the number of features substantially exceeds the sample size, the computed integrated factors exhibit high variability across different runs and MOFA+ robustness and performance tend to be lower than SNF. In contrast, NEMO is effective when all patient pairs share at least one common data view. When this condition is violated, NEMO may produce unreliable similarity estimates, which compromises the quality of the integrated patient similarity network. This limitation makes NEMO less robust in clinical settings where missing data patterns are highly heterogeneous. On the other hand, despite its success in various clinical applications, including those with limited sample sizes, SNF is not equipped to handle partial datasets. Supplementary Section S1 summarizes the state-of-the-art (SOTA) of partial data fusion techniques.

To overcome these limitations we introduce miss-SNF, a novel, task-agnostic data fusion approach specifically designed to integrate partial datasets. miss-SNF extends the non-linear message-passing strategy of SNF to reconstruct missing pairwise similarities within the combined PSNs. Unlike previous methods, miss-SNF can integrate missing data even when no data source is shared among different patient subsets, and it can simultaneously handle multiple sources that are entirely missing. Moreover, the integration process is independent of downstream tasks, making it applicable to both unsupervised (e.g. patient stratification) and supervised (e.g. diagnosis or outcome prediction) applications. While several PSN-based data fusion algorithms have been designed as an extension of SNF, miss-SNF is the first to adapt the SNF diffusion process for integrating partial datasets. A preliminary version of the algorithm has been presented at an international conference (Gliozzo *et al.* 2023).

## 2 Methods

miss-SNF leverages the message-passing mechanism of the SNF algorithm to combine a “local” PSN, $S^{\left(s\right)}$, which captures local neighborhood similarities between patients for data source *s*, with a “global” PSN, $P^{\left(v\right)}$, that represents global similarities among all patients for a different data source *v*. This integration is performed according to the following updating equation:

(1) $$P_{t+1}^{\left(s\right)}=S^{\left(s\right)}\;P_{t}^{\left(v\right)}{\left(S^{\left(s\right)}\right)}^{⊺}$$

Both $S^{\left(s\right)}$ and $P^{\left(s\right)}$ are n×n matrices, where *n* is the number of patients and are constructed using $W^{\left(s\right)}$, that represents a normalized exponential Euclidean similarity between patients according to the data source *s*. A detailed presentation of the SNF algorithm is provided in Supplementary Section S2 and Supplementary Fig. S8.

A problem with the message-passing strategy of Equation (1) is that, in case of completely missing data for source *s* for a subset of patients we cannot leverage the cross diffusion between different sources of data *s* and *v*, since no data are available for that subset of patients. Naive solutions, e.g. setting to 0 the feature values for completely missing samples, lead to inconsistent patient similarities, since the similarity would be computed between the 0 vector and the omics profiles of the other patients.

Instead, miss-SNF provides a solution by tweaking the three matrices $W^{\left(s\right)}$, $P^{\left(s\right)}$, and $S^{\left(s\right)}$ to safely add some data even for samples with completely missing data sources, *s*, in order to enable the message-passing procedure. This can be accomplished by observing that every patient, independently to any data source, is inherently similar to himself. Therefore, by introducing a self-loop for patients lacking data in source *s*, i.e. by leveraging patients auto-similarity, we enable the message-passing process to update the global similarity matrix $P^{\left(s\right)}$.

We propose different strategies for constructing these self-loops and managing similarities:

***miss-SNF one***: assigns a self-loop with weight 1 while setting all similarities with other patients to 0. This approach assumes that patients are initially similar only to themselves.

***miss-SNF equidistant***: assigns a self-loop with weight 0.5 and small, equal similarities to all other patients. This reflects both a strong self-similarity and an initial weak similarity with others.

Note that both *miss-SNF one* and *miss-SNF equidistant* allow the recovery of global similarities for patients with completely missing data by leveraging similarities from other data sources through the message-passing mechanism.

***miss-SNF zero***: sets self-loops to 0, effectively ignoring partial samples. While this still enables message passing, it simply ignores missing data and does not allow the recovery of missing edges for the missing data sources.

In the rest of this section, we introduce the mathematical details of the proposed miss-SNF strategies.

***miss-SNF one*** introduces a unique self-similarity (self-loop) for the partial sample *i* in all the similarity matrices of source *s* to allow message passing even when we have a completely missing source *s* for a patient *i*. This is achieved by setting:

$$W^{\left(s\right)}(i,i)=P^{\left(s\right)}(i,i)=S^{\left(s\right)}(i,i)=1,$$

and

$$W^{\left(s\right)}(i,j)=P^{\left(s\right)}(i,j)=S^{\left(s\right)}(i,j)=0\;\forall \;i\neq j.$$

Expanding Equation (1) to focus on the update of source *s* for two patients *i* and *j*, where we have complete missing source data *s* for patient *i*, the update formula of $P^{\left(s\right)}(i,j)$ for patients *i* and *j* becomes:

(2) $$P_{t+1}^{\left(s\right)}(i,j)=\sum_{k\in N_{i}}\sum_{l\in N_{j}}S^{\left(s\right)}(i,k)\;P_{t}^{\left(v\right)}(k,l)\;S^{\left(s\right)}(j,l)$$

where $N_{i}$ and $N_{j}$ are the *K*-nearest neighborhood of, respectively, *i* and *j* in *s*. Under this setting, we have two possible different cases for computing the updated value for $P_{t+1}^{\left(s\right)}(i,j)$ (Equation (2)):

1. when k≠i
 
  $$P_{t+1}^{\left(s\right)}(i,j)=\sum_{k\in N_{i}}\sum_{l\in N_{j}}S^{\left(s\right)}(i,k)P_{t}^{\left(v\neq s\right)}(k,l)S^{\left(s\right)}(j,l)=0$$
2. when k=i 
$$\exists l\;\text{s.t.}\;S^{\left(s\right)}(j,l)>0\;\text{and}\;P_{t}^{\left(v\neq s\right)}(i,l)>0\Rightarrow$$
   
  $$P_{t+1}^{\left(s\right)}(i,j)=\sum_{l\in N_{j}}S^{\left(s\right)}(i,i)P_{t}^{\left(v\neq s\right)}(i,l)S^{\left(s\right)}(j,l)>0$$

Hence Equation (2) becomes:

$$\begin{array}{c} P_{t+1}^{\left(s\right)}(i,j)=\sum_{k\in N_{i}}\sum_{l\in N_{j}}S^{\left(s\right)}(i,k)\;P_{t}^{\left(v\right)}(k,l)\;S^{\left(s\right)}(j,l)=\\ =\sum_{l\in N_{j}}S^{\left(s\right)}(i,i)\;P_{t}^{\left(v\right)}(i,l)\;S^{\left(s\right)}(j,l)=\\ =\sum_{l\in N_{j}}P_{t}^{\left(v\right)}(i,l)\;S^{\left(s\right)}(j,l) \end{array}$$

In other words, we have a contribution to the missing $P_{t+1}^{\left(s\right)}(i,j)$ when *i* and *j* have a common neighbor *l* in, respectively, the “global” network $P^{\left(v\right)}$ of the other source and in the “local” network $S^{\left(s\right)}$ for the partial source *s*. This implies that we can populate $P^{\left(s\right)}(i,j)$ also when data are missing for source *s*. Supplementary Figure S8 provides a pictorial description of the miss-SNF algorithm.

If the dataset contains m>2 sources, Equation (2) becomes:

$$\begin{array}{c} P_{t+1}^{\left(s\right)}(i,j)=\sum_{l\in N_{j}}\sum_{k\in N_{i}}S^{\left(s\right)}(i,k)\;{\bar{P}}_{t}^{\left(v\neq s\right)}(k,l)\;\;S^{\left(s\right)}(j,l)=\\ =\sum_{l\in N_{j}}S^{\left(s\right)}(i,i)\;{\bar{P}}_{t}^{\left(v\neq s\right)}(i,l)\;S^{\left(s\right)}(j,l)=\\ =\sum_{l\in N_{j}}{\bar{P}}_{t}^{\left(v\neq s\right)}(i,l)\;S^{\left(s\right)}(j,l) \end{array}$$

where ${\bar{P}}_{t}^{\left(v\neq s\right)}(i,l)=\frac{\sum_{v\neq s}P_{t}^{\left(v\right)}(i,l)}{m-1}$ is the global average similarity between patients *i* and *l* across data sources different from *s*.

According to the above equation $P_{t+1}^{\left(s\right)}(i,j)$ is reconstructed if patients *i* and *j* share common neighbors, respectively, in the global network ${\bar{P}}^{v\neq s}$ and in the local network $S^{\left(s\right)}$.

***miss-SNF zero*** simply ignores partial samples, $x_{i}$, in the diffusion process by setting $W^{\left(s\right)}(i,j)=P^{\left(s\right)}(i,j)=S^{\left(s\right)}(i,j)=0,\forall j$. By applying computations similar to those shown for miss-SNF *one*, it is straightforward to see that $P_{t+1}^{\left(s\right)}(i,j)=0\;\forall j$ (Supplementary Fig. S8).

In this case, the integrated similarity $P^{\left(c\right)}$ is computed as

$$P^{\left(c\right)}=\left(\sum_{v=1}^{m}P^{\left(v\right)}\right)⊙M,$$

where ⊙ is a pointwise multiplication, and M is a matrix of the same dimension of P where M(i,j) is the reciprocal of the number of sources containing information for both *i* and *j*. In this way we obtain a “consensus” $P^{\left(c\right)}$ that averages with respect to the actually available data source for each patient.

***miss-SNF equidistant*** sets the self-loop on each partial patient *i* to $W^{\left(s\right)}(i,i)=0.5$ and assumes all the other samples are equally distant (Supplementary Fig. S8). This is achieved by setting $W^{\left(s\right)}(i,j)=0.5/(n-1),\forall j\neq i$, where *n* is the number of patients. The same is done for $P^{\left(s\right)}$ and $S^{\left(s\right)}$. In this way, patient *i* is more similar to himself than to the other patients, and each patient is initially equally similar to the other patients, which is a fair assumption considering the complete lack of information about source *s* for patient *i*.

***miss-SNF random*** sets random uniformly distributed similarities for sample *i* in $W^{\left(s\right)}$, $P^{\left(s\right)}$, and $S^{\left(s\right)}$. This method is a sort of baseline to test whether miss-SNF performance is really influenced by the initial values setting of the partial sources.

## 3 Experimental evaluation

### 3.1 Datasets

miss-SNF is evaluated on nine multi-omics datasets from TCGA (Hutter and Zenklusen 2018): BLadder urothelial Carcinoma (BLCA); BReast infiltrating ductal CArcinoma (BRCA1) and BReast infiltrating lobular CArcinoma (BRCA2); KIdney Renal Clear cell carcinoma (KIRC); LUng ADenocarcinoma (LUAD); LUng Squamous Cell carcinoma (LUSC); PRostate ADenocarcinoma (PRAD); OVarian serous cystadenocarcinoma (OV); SKin Cutaneous Melanoma (SKCM). Each dataset comprises four data sources: miRNA, mRNA, protein expression, and DNA methylation. Clinical variables are downloaded and used to evaluate miss-SNF on unsupervised clustering.

The TCGA-CDR dataset (Liu *et al.* 2018) further provides standardized clinical features derived from the TCGA program, addressing the incomplete availability of outcome and treatment information and the relatively short follow-up durations. The curated information about the patients’ Overall Survival (OS) and Progression Free Interval (PFI) have been used to evaluate miss-SNF in supervised classification tasks.

Before any further analysis, the multi-omics datasets were pre-processed to filter variables mainly carrying noise or highly redundant information. After *Z*-score standardization, we reduced the effect of the small-sample-size problem by dimensionality reduction of each view. To this aim, according to Gliozzo *et al.* (2025), we chose the randomized principal component analysis (Erichson *et al.* 2019) and set the dimension of the latent space by computing the view intrinsic dimensionality (id), via the *two-nn* (Facco *et al.* 2017) algorithm, nowadays one of the most effective id estimators.

An exhaustive description of the TCGA datasets, their descriptive statistics, the clinical variables we downloaded, and the details of the data pre-processing can be found in Supplementary Section S3 and Supplementary Table S5.

Our analysis is focused on cancer TCGA data, but to show the applicability of miss-SNF also to multi-omics non-cancer data, we performed classification experiments using the ROSMAP dataset, which includes three omics types (mRNA, methylation, and miRNA expression) for 182 Alzheimer’s disease patients and 169 normal control patients (Wang *et al.* 2021).

### 3.2 Experimental setup

We evaluated miss-SNF in three main experimental settings characterized by partial (completely missing) data: (a) data recovery capability, i.e. reconstructing global patient similarity; (b) patients cluster recovery capability; (c) recovery of phenotype/outcome prediction capabilities.

To objectively assess and compare the information recovery reliability of the different miss-SNF algorithms, all the nine datasets were filtered to retain only their complete versions, excluding partial cases. This allowed the application of standard SNF, which served as a gold standard for comparison with miss-SNF applied to their randomly amputated counterparts.

To this end we evaluated miss-SNF under varying partial-missingness conditions, by randomly amputating each dataset to remove x% (x∈{10,20,30,40,50}) of samples from each data source. The removal process was performed independently for each data source, but in such a way as to ensure that at least one data source remained available for each patient. To ensure statistical significance, h=10 randomly amputated datasets were generated for each amputation percentage. Missing data patterns for all cancers and the ROSMAP Alzheimer’s disease dataset are present in Supplementary Figs S9–S18.

Note that our choice to simulate missing data by amputating multiple data sources at the same time may introduce additional complexity compared to the simpler scenario where only a single source is amputated. However, this design more accurately reflects clinical settings, where it is common for several data sources to be completely missing for different subsets of patients. Demonstrating the robustness of miss-SNF under this challenging multi-source missingness scenario guarantees its effectiveness in the simpler case of single-omic missingness as well.

Regarding SNF and miss-SNF hyperparameters, all experiments were conducted with T=100 iterations for all models except miss-SNF *one*, where T=1000 was used. This adjustment was based on convergence analysis results, which indicated slower convergence for miss-SNF *one* (see Supplementary Section S4 for details).

In our experiments, we compared miss-SNF with MOFA+ and NEMO algorithms, two state-of-the-art methods for the integration of partial datasets.

**Figure 1.**
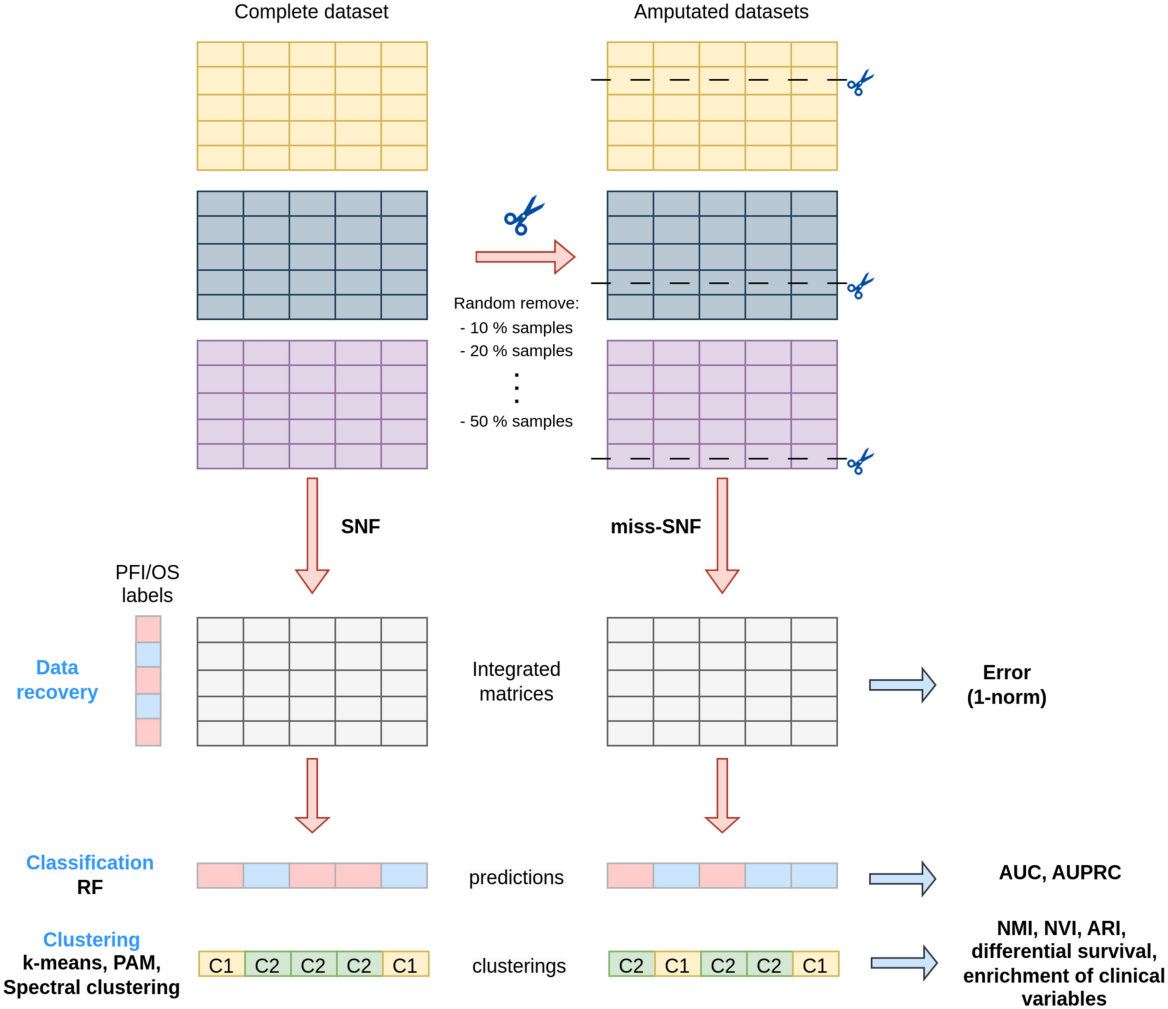
Experimental setup for miss-SNF evaluation.

Statistical comparison was performed by computing win–tie–loss tables by paired Wilcoxon signed rank test (details in Supplementary Section S5). A sketch of the evaluation schema is shown in Fig. 1.

### 3.3 Results

#### 3.3.1 Recovery of missing pairwise similarities

**Figure 2.**
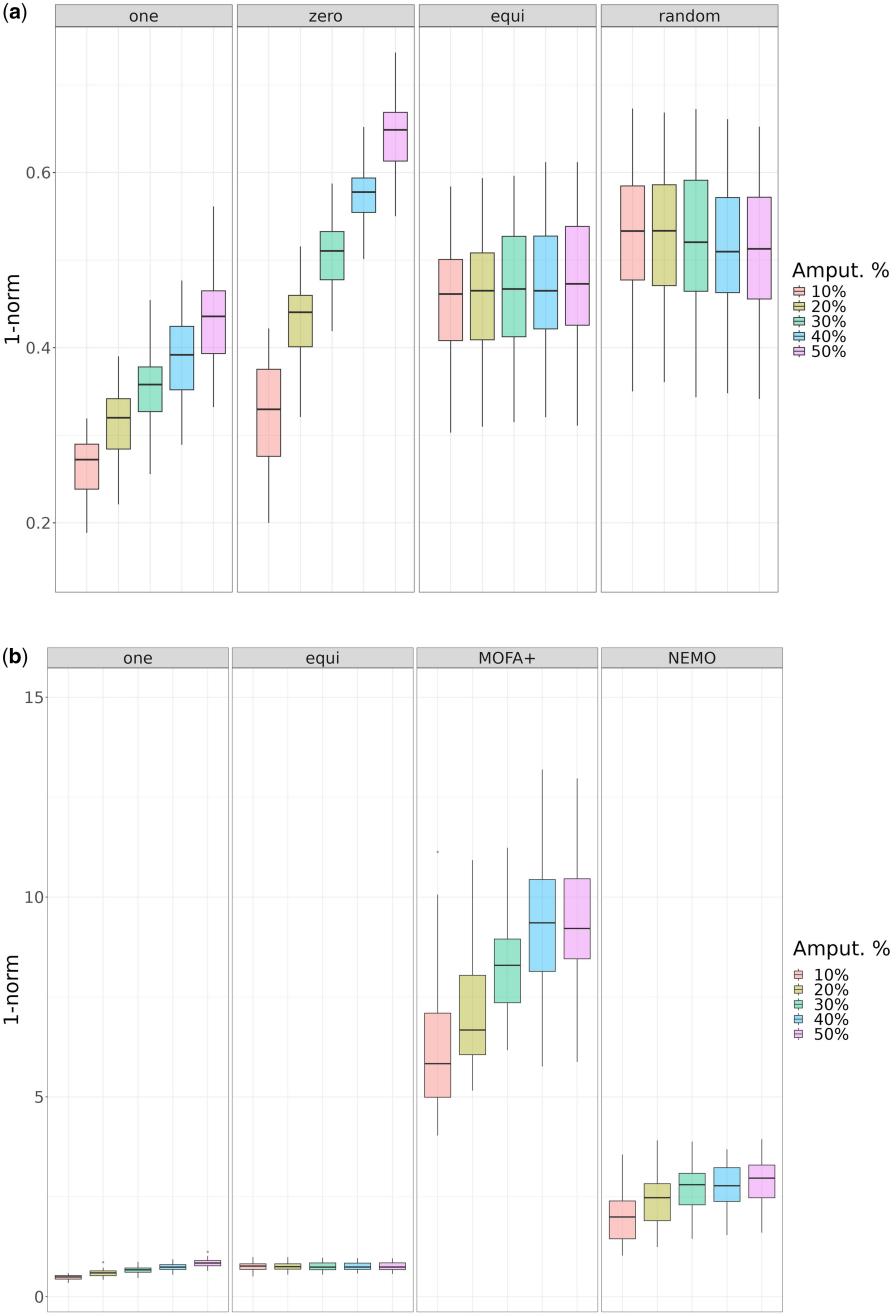
Comparison of the distribution of the 1-norm difference between amputated and complete datasets for different amputation ratios. (a) Comparison of miss-SNF one, zero, equidistant, and random; (b) Comparison between miss-SNF one, equidistant, MOFA+ and NEMO (min–max scaling normalization of PSN similarity values was applied to assure a fair comparison between different integration methods).

To evaluate the performance of miss-SNF in recovering information for partial samples, we applied SNF (Wang *et al.* 2014) to integrate the complete datasets and miss-SNF to integrate their amputated counterparts. Integration quality was assessed on each dataset by calculating the “reconstruction error” by means of the 1-norm distance between the fused PSN from the complete dataset (via SNF) and the 10 randomly generated amputated datasets (via miss-SNF) at each amputation percentage (the reconstruction error is computed using the formula $||P_{\mathrm{comp}}^{\left(c\right)}-P_{\mathrm{amp}}^{\left(c\right)}|{|}_{1}$, where $P_{\mathrm{comp}}^{\left(c\right)}$ and $P_{\mathrm{amp}}^{\left(c\right)}$ are the integrated matrices obtained from the complete and amputated datasets, respectively). Figure 2a shows the distribution of the reconstruction errors obtained for increasing percentages of partial examples. As expected, all versions of miss-SNF, except miss-SNF *random*, show increasing difficulty (increasing reconstruction errors) in integrating data sources as the proportion of missingness rises. The behavior of miss-SNF *random* can be attributed to its random similarity assignment for partial samples. This approach introduces bias into the integrated matrix by incorporating incorrect similarities during the diffusion process, resulting in noisy fused matrices that remain largely unaffected by the proportion of missing data.

Overall, miss-SNF *one* and *equidistant* achieve the lowest reconstruction errors. In contrast, as expected, miss-SNF *zero* fails to recover information for partial samples, especially when the percentage of amputated data increases, since it completely disregards these samples during the diffusion process. Win–tie–loss plots in Supplementary Fig. S4 show that miss-SNF *one* achieves the best results (one-sided paired Wilcoxon test, α=0.05), followed by miss-SNF *equidistant*. Boxplots of the reconstruction error for individual cancers are available in Supplementary Fig. S5.

Driven by these results, in the following we limit our analysis to miss-SNF *one* and miss-SNF *equidistant* and we report in Supplementary Fig. S6 the reconstruction error boxplots for miss-SNF *one* and *equidistant* with the ROSMAP dataset.

#### 3.3.2 Compared to SOTA methods, miss-SNF is more effective in similarity recovery

Figure 2b shows the reconstruction error distributions computed across all datasets by miss-SNF *one*, *equidistant*, MOFA+, and NEMO. miss-SNF *one* and *equidistant* have the lowest reconstruction error across all amputation percentages with a slight increase for higher proportions of missing values. NEMO exhibits higher error values while the worst approach is MOFA+ with significant errors that consistently increase as the amount of partial samples increases. The win–tie–loss tables summarizing the results of one-sided paired Wilcoxon tests (Supplementary Fig. S20 and Supplementary Table S6) confirm that miss-SNF (*one* and *equidistant*) outperforms NEMO and MOFA+.

#### 3.3.3 Clustering experiments

We conducted clustering experiments using SNF (on complete datasets), miss-SNF (on amputated datasets), MOFA+, and NEMO (on both complete and amputated datasets). Three clustering methods were employed: (1) *k*-means (Hastie *et al.* 2009) algorithm applied both to the fused PSN (from now on *k*-means (similarities)), and to the factors in the case of MOFA+ (from now on *k*-means (factors)); (2) spectral clustering (SP), using both the algorithm described in Ng *et al.* (2001) (SP-sClust) and the version provided by NEMO’s authors (SP-SNFtool, applied exclusively to PSNs integrated by NEMO); (3) partitioning around medoids (PAM) (Kaufman and Rousseeuw 1990), applied to the fused PSN (PAM (similarities)), and to the factors in the case of MOFA+ (PAM (factors)).

By combining each partial data fusion approach (miss-SNF *one* and *equidistant*, MOFA+, and NEMO) with each of the clustering methods, we derived a total of 15 distinct partial data fusion + clustering approaches (see the full list in Supplementary Table S7).

To avoid bias in determining the optimal number of clusters, as recommended by Duan *et al.* (2021), we evaluated clustering results for a range from 2 to 10 clusters. Additional details regarding the experimental setup for clustering experiments are available in Supplementary Section S7.

#### 3.3.4 Comparison between clusters obtained from complete and amputated data

Our initial analysis focused on comparing clusters derived from complete datasets with those generated from partial datasets.

Supplementary Figures S21–S23 show the computed Normalized Mutual Information (NMI), Normalized Variation of Information (NVI), and Adjusted Rand Index (ARI) (You 2021). Win–tie–loss results in Supplementary Tables S7–S9 summarize the statistical comparison of the computed NMI, NVI, and ARI values. Independently from the considered metric, NEMO followed by SP (or *k*-means) is the best-performing combination, followed by miss-SNF *one* (any clustering method). MOFA+ has the lowest results for all metrics. With PAM algorithm miss-SNF outperforms both NEMO and MOFA+ in cluster recovery. Focusing on the best two combinations of data fusion and clustering method (NEMO + SP-SNFtool and miss-SNF *one* + *k*-means), we can see a steady and expected decrease in metric values (increase for NVI) as the percentage of partial data increases.

#### 3.3.5 Cluster enrichment and differential survival analysis

As a further comparison, we assessed the clinical relevance of the identified clusters with amputated data using a set of clinically relevant variables by counting the number of enriched variables per cluster (more details in Supplementary Section S7.1).

**Figure 3.**
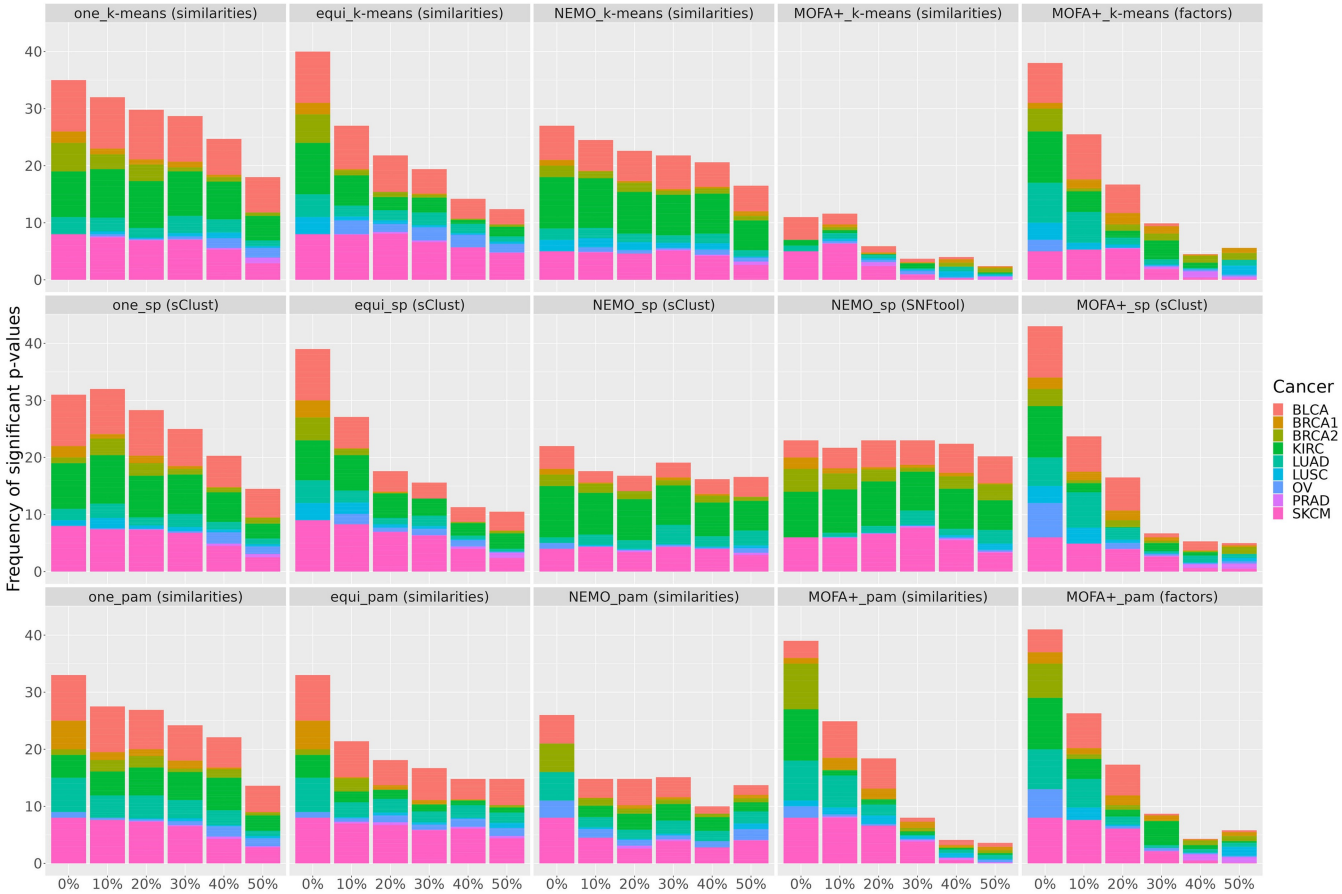
Comparison of the frequency for which a significant overall survival (log-rank test α=0.05) is registered in clusters obtained by different integration methods and clustering algorithms with increasing amputation data (*x*-axis) across different types of cancer. First column refers to miss-SNF one, the second to miss-SNF equidistant, the third to NEMO, the fourth to MOFA+ (using similarities), and the fifth to MOFA+ (using factors). First row refers to *k*-means clustering, the second to spectral clustering and the third to PAM. Colors corresponds to different types of cancer.

We assessed the ability of considered methods to identify clusters with differential survival, by applying the log-rank test with a 95% confidence level (α=0.05). For each amputation percentage, all 10 randomly amputated versions of each cancer dataset were clustered, producing 10 log-rank *P*-values. These *P*-values were aggregated by calculating the proportion of significant tests (*P*-value <α) across the 10 repetitions. The results of the survival analysis are presented in Fig. 3 and summarized in the win–tie–loss Supplementary Table S11. miss-SNF *one* consistently emerges as the best-performing method, followed by NEMO combined with spectral clustering and *k*-means clustering. In contrast, MOFA+ shows the lowest performance, regardless of the clustering technique applied.

Furthermore, miss-SNF *one* consistently produces clusterings enriched with more clinical variables, regardless of the clustering method employed. Conversely, MOFA+ demonstrates the lowest performance among the tested approaches (Supplementary Table S10 and Supplementary Fig. S24). Among the top-performing methods, Supplementary Fig. S24 reveals a gradual decline in the number of enriched variables as the percentage of partial samples increases for miss-SNF *one*, irrespective of the clustering technique used.

The final evaluation aimed to further assess the ability of the partial data fusion + clustering approaches to identify clinically enriched clusters after selecting the optimal number of clusters using two methods: (I) the modified eigengap method (Rappoport and Shamir 2019), a standard approach for determining the number of clusters based on spectral clustering properties; (II) the “Clin-Surv” strategy, an approach we propose that selects the optimal number of clusters as the configuration yielding the highest number of clinically enriched variables. In case of ties, the configuration with the lowest log-rank test *P*-value was chosen. The comparison across evaluation metrics (i.e. NMI, NVI, ARI) yield results consistent with those obtained when all cluster numbers were considered (Supplementary Tables S12–S14 and Supplementary Figs S25–S27). More interestingly, the analysis showed that miss-SNF *one* and NEMO, both combined with *k*-means, consistently achieved the largest proportions of clinically enriched variables (Supplementary Table S15 and Supplementary Fig. S28). By contrast, all variants of MOFA+ exhibited the lowest performance.

The results obtained from log-rank tests are more difficult to interpret due to the high number of ties in the win–tie–loss (Supplementary Table S16 and Supplementary Fig. S29). It is however clear that miss-SNF and NEMO, both followed by *k*-means are again the best-performing models.

#### 3.3.6 Classification experiments

**Figure 4.**
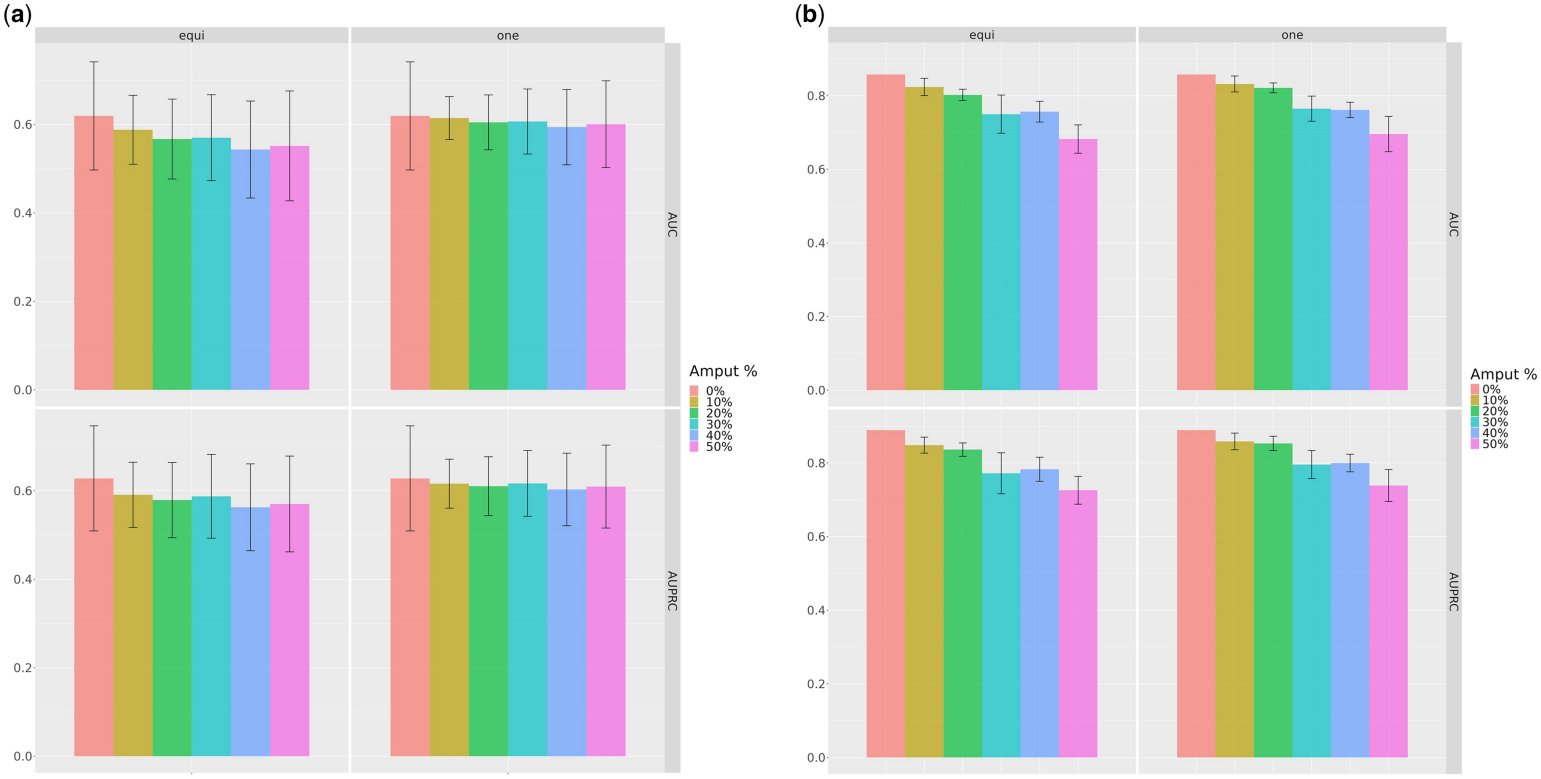
Classification results using SNF on complete dataset and miss-SNF with amputated datasets. (a) Performance for overall survival (OS) prediction (TCGA datasets); (b) performance for Alzheimer’s disease prediction (ROSMAP dataset). Colors refer to different amputation percentages.

We conducted classification experiments using miss-SNF *one* and *equidistant* (Section 3.3) to compare results obtained with full and amputed data sets. Experiments were performed by applying Random Forest (RF) (Breiman 2001) classifier on the integrated matrices computed over each TCGA dataset and the ROSMAP dataset. Following TCGA-CDR recommendations, supervised classification over the TCGA datasets was applied to predict OS and PFI events. Unbiased classification performance on TCGA datasets was evaluated by using 10 random stratified holdouts (90% training set, 10% test set) and by computing the Area Under the ROC Curve (AUC) and Area Under the Precision-Recall Curve (AUPRC) as evaluation metrics. Feature selection and hyper-parameter tuning were performed using 10 internal holdouts (additional details in Supplementary Section S8). Results were summarized by computing the average and pooled standard deviation (Moore *et al.* 2021) across the 10 amputations randomly generated for each amputation percentage, folds and datasets. Classification performance on ROSMAP dataset were evaluated using RF with default hyperparameters on a stratified split (70% training set, 30% test set). Figure 4 and Supplementary Fig. S19 compare the AUC and AUPRC obtained by miss-SNF. The classification metrics only show a low decrease with the random amputation of increasing percentages of samples in both tasks, confirming the robustness and reliability of miss-SNF *one* and *equidistant*. With TCGA datasets miss-SNF *one* exhibits a more gradual decline in performance compared to miss-SNF *equidistant*, highlighting its superior ability to reconstruct missing information.

## 4 Discussion

In PSN multimodal data integration it is quite common that not all the sources of omics or clinical data are available for all patients. miss-SNF provides a simple strategy to recover missing similarities for subsets of patients, allowing the construction of global similarity networks P even with patients having completely missing data for some data sources.

Thorough evaluations on nine multi-omics cancer datasets with increasing levels of missing data (10%–50%) showed the following strengths of miss-SNF *one* and *equidistant*.

**Robust recovery and integration:** when compared to SNF application on the complete datasets, miss-SNF *one* and *equidistant* excelled at similarity recovery and integration, outperforming SOTA methods like MOFA+ and NEMO.

**Theoretically grounded strategy:** unlike some other SOTA methods—e.g. NEMO, that apply a smart workaround to deal with missing data, miss-SNF directly leverages patient neighborhoods, making it theoretically grounded and versatile.

**Effectiveness in unsupervised clustering:** clusters generated with miss-SNF *one* on partial datasets closely matched those from full datasets while identifying clinically relevant patient groups. miss-SNF *one* outperformed competing methods in enriching clinical variables and achieving significant log-rank tests, particularly when the optimal cluster count was selected via the proposed *clin-surv* method.

**Supervised learning potential:** using RFs, we showed that the PSN computed by miss-SNF *one* and *equidistant* using partial data achieved performance close to that obtained when the complete full data set is available, highlighting their suitability for inductive and also transductive learning tasks (Zhu and Ghahramani 2002, Zhu *et al.* 2003, Valentini *et al.* 2014), crucial for multi-omics datasets with scarce manual annotations (Cappelletti *et al.* 2023).

**Advantages over SOTA methods:** unlike techniques tailored to specific tasks (e.g. clustering with MaDDA (Vitali *et al.* 2018) or SUMO (Sienkiewicz *et al.* 2022), classification with DeepIMV (Lee and van der Schaar 2021) or MOGDx (Ryan *et al.* 2024)), the miss-SNF algorithm produces a general-purpose integrated matrix suitable for both supervised and unsupervised tasks. miss-SNF is flexible and modular in the sense that does not depend on specific clustering or classification algorithms: the generated integrated PSNs can be analysed with any clustering or supervised classification method. It is particularly advantageous for biomedical datasets, usually characterized by low sample cardinality, where deep learning methods struggle due to sample size limitations.

In summary, miss-SNF *one* stands out as a robust, flexible solution for partial data fusion, excelling in recovery, clustering, and classification tasks while adapting to real-world challenges of incomplete data. To the best of our knowledge, this is the first algorithm to extend SNF message-passing process for partial dataset integration, setting a new benchmark for multi-omics data analysis (Gliozzo *et al.* 2023).

While we performed our tests on multi-omics data, we note that miss-SNF inherits the advantages of SNF; it is therefore a versatile tool that may be used to integrate other data-types, whenever proper pairwise similarity metrics are used. Additionally, it does not rely on *a priori* biological knowledge, making it suitable for combining diverse patient data, such as clinical records or imaging features, and adaptable across various research fields beyond omics.

Although we showed the effectiveness of miss-SNF for both unsupervised and supervised tasks, this work has some limitations. miss-SNF *one* is the best-performing version of our algorithm; however, it exhibits slower convergence when compared to both SNF and the other miss-SNF variants, which may limit its efficiency for large-scale or time-sensitive applications. Furthermore, while our tests on supervised classification showed that using partial data miss-SNF can obtain results close to that achievable when full data are available, results on TCGA datasets were still suboptimal probably due to the inherent difficulty of the chosen tasks and the complexity of the used datasets. On the other hand, the predictions achieved on Alzheimer’s disease diagnosis show that miss-SNF can achieve better results when more informative data are available.

## Supplementary Material

btaf150_Supplementary_Data

## Data Availability

The Cancer Genome Atlas (TCGA) legacy data are available to download from https://gdac.broadinstitute.org/runs/stddata__2016_01_28/. TCGA-CDR dataset is included in the “Supplemental information” of Liu *et al.* (2018) manuscript. The ROSMAP dataset is available from the MOGONET (Wang *et al.* 2021) GitHub repository.

## Code availability

miss-SNF is publicly available from GitHub (https://github.com/AnacletoLAB/missSNF) or Zenodo (https://doi.org/10.5281/zenodo.14945615).
